# A new lower actinopterygian fish from the Upper Mississippian Bluefield Formation of West Virginia, USA

**DOI:** 10.7717/peerj.5533

**Published:** 2018-08-29

**Authors:** Kathryn E. Mickle

**Affiliations:** Jefferson College of Life Sciences, Thomas Jefferson University, Philadelphia, PA, United States of America

**Keywords:** Lower actinopterygian, Carboniferous, Mississippian, Palaeoniscoid, Fossil fish

## Abstract

The Upper Mississippian Bluefield Formation of the Mauch Chunk Group in southeastern West Virginia is known for its preservation of a variety of invertebrate taxa and early tetrapod trackways, but no lower actinopterygian remains have been formally described from these Carboniferous rocks. Here, the first lower actinopterygian fish is described from the Bluefield Formation of West Virginia. This fish is represented by a nearly complete articulated specimen with a three-dimensional snout and an unobstructed view of the gular and branchiostegal region. This new taxon is defined by a unique set of characters, which include features of the snout, circumorbital series, cheek, and operculo-gular region. These features make this fish different and distinct from previously described Carboniferous fishes. Some of the morphological features of note include the presence of a distinct lacrimal, premaxillary, ventral rostral and dorsal rostral bones, a narrow infraorbital ventral to the orbit, and a large crescent shaped infraorbital that contacts a single dermosphenotic. There is an anteriorly inclined hatchet-shaped preoperculum and six small suborbital bones anterior to the expanded region of this bone that filling the space between the preoperculum, dermosphenotic, and infraorbital. Posterior to the preoperculum, there is a single wedge-shaped dermohyal and a series of three rectangular anteopercular bones. The anteopercular bones extend halfway down the anterior border of the rectangular operculum. A median gular, two pairs of lateral gulars, and at least eight branchiostegal rays are present. The heterocercal caudal fin is deeply cleft and inequilobate. The scales have pectinated posterior margins and bear diagonal ridges of ganoine. The description of this new taxon represents the first actinopterygian and the first vertebrate body fossil described from the Bluefield Formation and the second actinopterygian taxon described from the Mauch Chunk Group in West Virginia.

## Introduction

### Importance of descriptive work

The body plan of modern fishes is thought to have originated within a grouping of lower actinopterygian fishes collectively referred to as palaeoniscoids (Gardiner, 1984; Zhu & Schultze, 2001). Though this makes Paleozoic lower actinopterygians important fishes, this is overshadowed by the fact that the systematics of these fishes has been, and still is, a source of controversy. The majority of phylogenetic investigations into lower actinopterygian fishes have recovered palaeoniscoids as paraphyletic (See Cloutier & Arratia, 2004; Friedman & Blom, 2006; Mickle, Lund & Grogan, 2009). This is attributed to several reasons, one of which is the fact that even the largest analyses to date have only included a small portion of the diversity these fish present (Cloutier & Arratia, 2004; Mickle, Lund & Grogan, 2009). Accordingly, descriptions of new taxa that are represented by well-preserved specimens are vital to our understanding of these fishes in particular, and lower actinopterygians in general. Here, a single well-preserved fossil fish specimen that represents a new lower actinopterygian genus and species is described from the Bluefield Formation of West Virginia.

### Bluefield Formation

**Figure 1:**
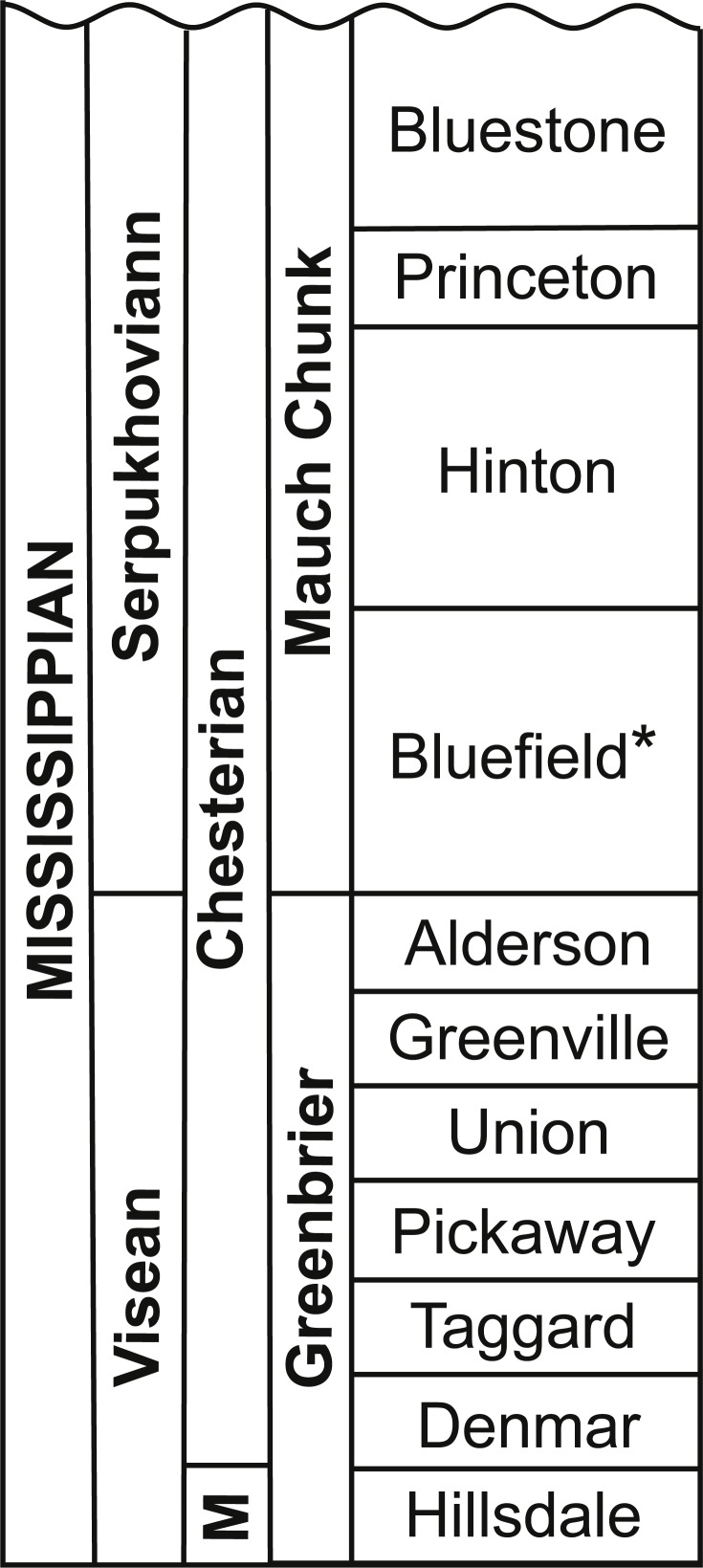
Stratigraphic column detailing the Mauch Chunk Group and the Bluefield Formation of West Virginia. Modified from Powell (2008, fig. 1A). Abbreviation: M, Meramecian. The Bluefield Formation is highlighted with an asterisk.

The Upper Mississippian Bluefield Formation is the basal-most formation in the Mauch Chunk Group in the Appalachian Basin (Maynard, Eriksson & Law, 2006). In southeastern West Virginia, the Mauch Chunk Group is divided into four formations, from the bottom to the top; the Bluefield, Hinton, Princeton, and Bluestone formations (Maynard, Eriksson & Law, 2006; Powell, 2008; Peck et al., 2009; here Fig. 1). The Bluefield Formation is Late Mississippian (Chesterian in local North American terminology) in age and has been correlated with the late Viséan to early Serpukhovian (Powell, 2008), or in European terminology, the basal-most portion of the Bluefield correlates with the upper Viséan and upper portions to the Namurian A (Beuthin & Blake, 2004; Maynard, Eriksson & Law, 2006; Zaton & Peck, 2013).

Early tetrapod trackways of the amphibian *Hylopus hamesi* have been described from the Bluefield Formation (Sundberg et al., 1990) which is known for an abundant and diverse invertebrate fauna (Cranston, 1990; Maynard, Eriksson & Law, 2006; Peck et al., 2009). Surprisingly, fossil fishes are absent from these Mississippian rocks. Though there is mention of the recovery of fish teeth and scales (Zaton & Peck, 2013), there have been no fishes described previously from the Bluefield Formation. There has been only one actinopterygian, the deep-bodied *Tanypterichthys pridensis*, from the Mauch Chunk Group, and specifically the Bluestone Formation in West Virginia (Weems & Windolph Jr, 1986). The new genus presented here is the first and only fish, and vertebrate body fossil, described from the Upper Mississippian Bluefield Formation in West Virginia.

#### Geological setting and paleoenvironment

The fish specimen described here was collected from the same locality as the *Hylopus* trackways (Sundberg et al., 1990) but specific information as to where in this locality is lacking. Because of this, the depositional environment of the entire locality is reviewed so that generalizations regarding what type of environment this fish lived in can be made.

**Figure 2:**
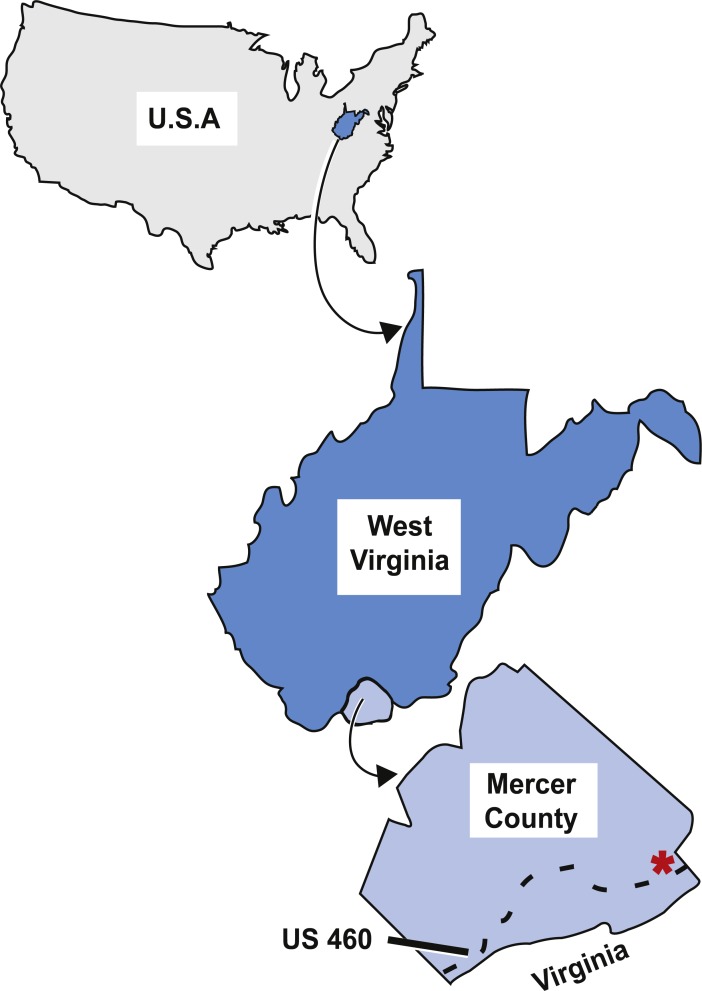
Map of the locality the type specimen was collected from. Asterisk indicates locality KUVP 155843 was collected from in Mercer County, West Virginia, dashed line indicates highway US 460. Figure modified from Peck et al. (2009, fig. 1).

As described by Sundberg et al. (1990), the tetrapod trackways, and subsequently the fish specimen, were collected from an outcrop off the north side of US 460 in West Virginia close to the West Virginia-Virginia state line (Fig. 2). This outcrop is on the overturned southeast limb of the Glen Lyn Syncline near Glen Lyn, Virginia (Whisonant & Schultz, 1986; Sundberg et al., 1990) and contains well exposed strata from the middle portion of the Bluefield Formation (Whisonant & Schultz, 1986; Sundberg et al., 1990). This middle portion is above the carbonate units of the underlying lower portion of the Bluefield Formation and Greenbrier Limestone, and below the terrigenous upper portion of the Bluefield Formation and Hinton Formations (Whisonant & Schultz, 1986). The middle portion of the Bluefield Formation is composed of interbedded limestone and siliciclastic layers and is thought to represent a change in the sedimentary regime from carbonate to terrigenous conditions in the Late Paleozoic Southern Appalachians (Whisonant & Scolaro, 1980; Whisonant & Schultz, 1986).

The strata at this locality preserve evidence of two major cycles of regression and fluctuating shore-related environments (Whisonant & Schultz, 1986). There are five distinct lithofacies within the first regressive cycle. The lowest strata in this cycle are poorly exposed and composed of ooidal and skeletal grainstones-packstones with uneven erosional bases and many pelmatozoan and bryozoan fossils (ibid.). This type of strata is proposed to have been deposited in shallow marine shoals (Whisonant & Scolaro, 1980; Whisonant & Schultz, 1986). Above this are calcareous shales and argillaceous wackestones-mudstones with blastoids, bryozoan *Archimedes* colonies, corals, and brachiopods (Whisonant & Schultz, 1986). These wackestones and mudstones are interpreted as shoreward calm, protected, lagoon-like waters (Whisonant & Scolaro, 1980; Whisonant & Schultz, 1986). Above this are silty sandstones with transported plant fragments, ripple marks, and desiccation cracks (Whisonant & Schultz, 1986). This lower sandstone is proposed to have been deposited in a lower to middle intertidal environment (Whisonant & Scolaro, 1980; Whisonant & Schultz, 1986). This lower sandstone layer is overlain by sandstones composed of medium to thick facies with transported plant fragments and ripple marks that represent shallow subtidal or intertidal environments (Whisonant & Scolaro, 1980; Whisonant & Schultz, 1986). Above the two sandstone units is a calcerous shales and siltstones facies with mudcracks, burrows, horizontal trails, and transported plants that represent a muddy middle to higher intertidal environment (Whisonant & Scolaro, 1980; Whisonant & Schultz, 1986). The first regressive cycle preserves the transition from shallow shoals to calm lagoonal conditions, to subtidal to intertidal environments (Whisonant & Schultz, 1986).

The second regressive cycle also contains five distinct lithofacies that preserve the fluctuations from a protected lagoonal environment, to ooidal shoals, to subtidal to intertidal environments (Whisonant & Schultz, 1986). At the base of this regressive cycle are mudstones and wackestones that represent calm lagoonal waters (Whisonant & Schultz, 1986). These are overlain by ooidal and skeletal grainstones-packstones representing shallow marine shoals (Whisonant & Schultz, 1986). Above the grainstones-packstones are silty sandstones from a lower to middle intertidal environment (Whisonant & Scolaro, 1980; Whisonant & Schultz, 1986). The silty sandstone layer is overlain by medium to thick sandstone facies that represent shallow subtidal or intertidal environments (Whisonant & Scolaro, 1980; Whisonant & Schultz, 1986). Overlaying this sandstone unit, and at the top of the regressive cycle, is a calcerous shales and siltstone facies that represent a muddy middle to higher intertidal environment. It is from these rocks that the tetrapod trackways were collected (Humphreville, 1981; Whisonant & Schultz, 1986; Sundberg et al., 1990).

Sundberg et al. (1990) conclude that the locality the trackways were collected from represents a non-marine fluvial or, at most, brackish estuarine habitat. This differs from previous interpretations of Whisonant & Scolaro (1980), Humphreville (1981), and Whisonant & Schultz (1986) that support an intertidal environment. Sundberg et al. (1990) based their reinterpretation on the absence of marine fossils and presence of terrestrial plant fragments and tetrapod trackways, a lack of salinity tolerance in recent amphibians, and a similar lack of evidence of such a tolerance in Carboniferous amphibians.

The lines of evidence used by Sundberg et al. (1990) for the reinterpretation are problematic for determining whether a locality was freshwater or marine. As pointed out by Schultze (2009), absence of evidence is not evidence for or against a particular locality being freshwater, brackish, or marine. The absence of marine fossils may mean the locality was freshwater, or it could mean that the marine forms did not preserve or were not collected (Schultze, 2009). Also pointed out by Schultze (2009), the presence of terrestrial plant fragments is not strong support for interpreting an environment as freshwater because fragments are not allochthonous to the aquatic environment and could be the result of transport from other areas. Lastly, Schultze (2009) cautions against inferring function in extinct forms from function in living forms. With these concerns in mind, the interpretations of Whisonant & Scolaro (1980), Humphreville (1981), and Whisonant & Schultz (1986) that the trackways were preserved in an intertidal environment are accepted here. The locality the fossil fish specimen was collected from preserves two cycles of regressions and transgressions and reflects the repeated transition from environments such as shallow marine shoals, calm lagoonal conditions, and intertidal environments (Whisonant & Scolaro, 1980; Humphreville, 1981; Whisonant & Schultz, 1986).

## Materials and Methods

### Material examined and methods

The new taxon is represented by a single specimen which is missing its counterpart. The specimen is housed in the Vertebrate Paleontology collection at the University of Kansas Natural History Museum (KUVP 155843), Lawrence, Kansas, USA.

The specimen was examined by stereomicroscopy. To aid in visualization of features, the specimen was viewed under 70% ethanol or shaded with magnesium oxide. Illustrations were prepared using a camera lucida and digital illustrations were constructed using Adobe Photoshop and Illustrator programs. Photographs were taken with a Canon XSi Digital SLR camera with a macro lens. When possible, morphometric measurements and meristic counts were taken following the scheme of Lund & Poplin (1997).

The electronic version of this article in Portable Document Format (PDF) will represent a published work according to the International Commission on Zoological Nomenclature (ICZN), and hence the new names contained in the electronic version are effectively published under that Code from the electronic edition alone. This published work and the nomenclatural acts it contains have been registered in ZooBank, the online registration system for the ICZN. The ZooBank LSIDs (Life Science Identifiers) can be resolved and the associated information viewed through any standard web browser by appending the LSID to the prefix http://zoobank.org/. The LSID for this publication is: urn:lsid:zoobank.org:pub:0F2C9D33-9584-4739-B914-5C4C243E0B1F. The online version of this work is archived and available from the following digital repositories: PeerJ, PubMed Central and CLOCKSS.

### Nomenclature

Lower actinopterygian skull bones have been identified differently by various researchers (See Moy-Thomas & Bradley Dyne, 1938; Rayner, 1951; Pearson & Westoll, 1979; Gardiner, 1984; Long, 1988; Arratia & Cloutier, 1996; Poplin & Lund, 2002 for examples of different identifications and names of bones of the snout, and Mickle, 2015 for a review of terminology schemes). Here, the bones of the snout are identified following the criteria presented by Mickle (2012); Mickle (2015). The use of lacrimal (Mickle, 2012) is accepted over antorbital (Mickle, 2015) but it should be noted that the use of the term lacrimal in this work differs from the use of the same term by other researchers like Gardiner (1984), Long (1988), and Choo (2011). Table 1 provides a comparison between the names used to identify the bones of the snout in this new taxon and how equivalent bones have been identified in previously described Carboniferous fishes. The newly described taxon is compared to previously described Carboniferous fishes in the discussion portion of this paper. The bones of the snout of the comparative fishes have been reidentified here using the criteria of Mickle (2012); Mickle (2015)). The criteria of Poplin (2004) are used to identify the dermosphenotic bone. Scutes, fulcra, and other fin ray elements are identified using the criteria of Arratia (2008).

**Table 1:** Comparison of snout terminology. Details of how specific bones of the snout have been identified in this work in comparison to previous publications with equivalent bones. Bone names in the second column are in bold and italicized to stand out among the citations. It is also noted if a bone name used here has been used differently in other publications.

Identification in this work	Identification(s) for equivalent bones in previous publications
Lacrimal	***Antorbital*** (e.g., Lund & Melton, 1982; Taverne, 1996; Lund & Poplin, 2002; Poplin & Lund, 2002) but different from the use of ***lacrimal*** in other publications (e.g., Gardiner, 1984; Gardiner, 1984; Choo, 2011)
Ventral Rostral	***Rostral*** (e.g., Lund & Melton, 1982; Lund & Poplin, 2002; Poplin & Lund, 2002) and ***Inferior Rostral*** (e.g., Taverne, 1996)
Dorsal Rostral	***Postrostral*** (e.g., Lund & Melton, 1982; Lund & Poplin, 2002; Poplin & Lund, 2002) and ***Superior Rostral*** (e.g., Taverne, 1996)
Premaxilla	***Premaxilla*** (e.g., Lund & Melton, 1982; Taverne, 1996; Poplin & Lund, 2002; Lund & Poplin, 2002) but different from the use of ***premaxilla*** in Gardiner (1984) and others cited in Mickle (2012); Mickle (2015)

## Results

### Systematic paleontology

**Table:** 

OSTEICHTHYES Huxley, 1880
ACTINOTPERYGII Cope, 1871
*Bluefieldius* n. gen.
urn:lsid:zoobank.org:act:01FDF534-6985-4E62-9CAF-EAE6FB4795B1

**Diagnosis.** As for the type and only species

**Type and only Species.** *Bluefieldius mercerensis* n. gen. n. sp

**Etymology.** After the Bluefield Formation where the specimen was recovered from.

**Table:** 

**Figure 3:**
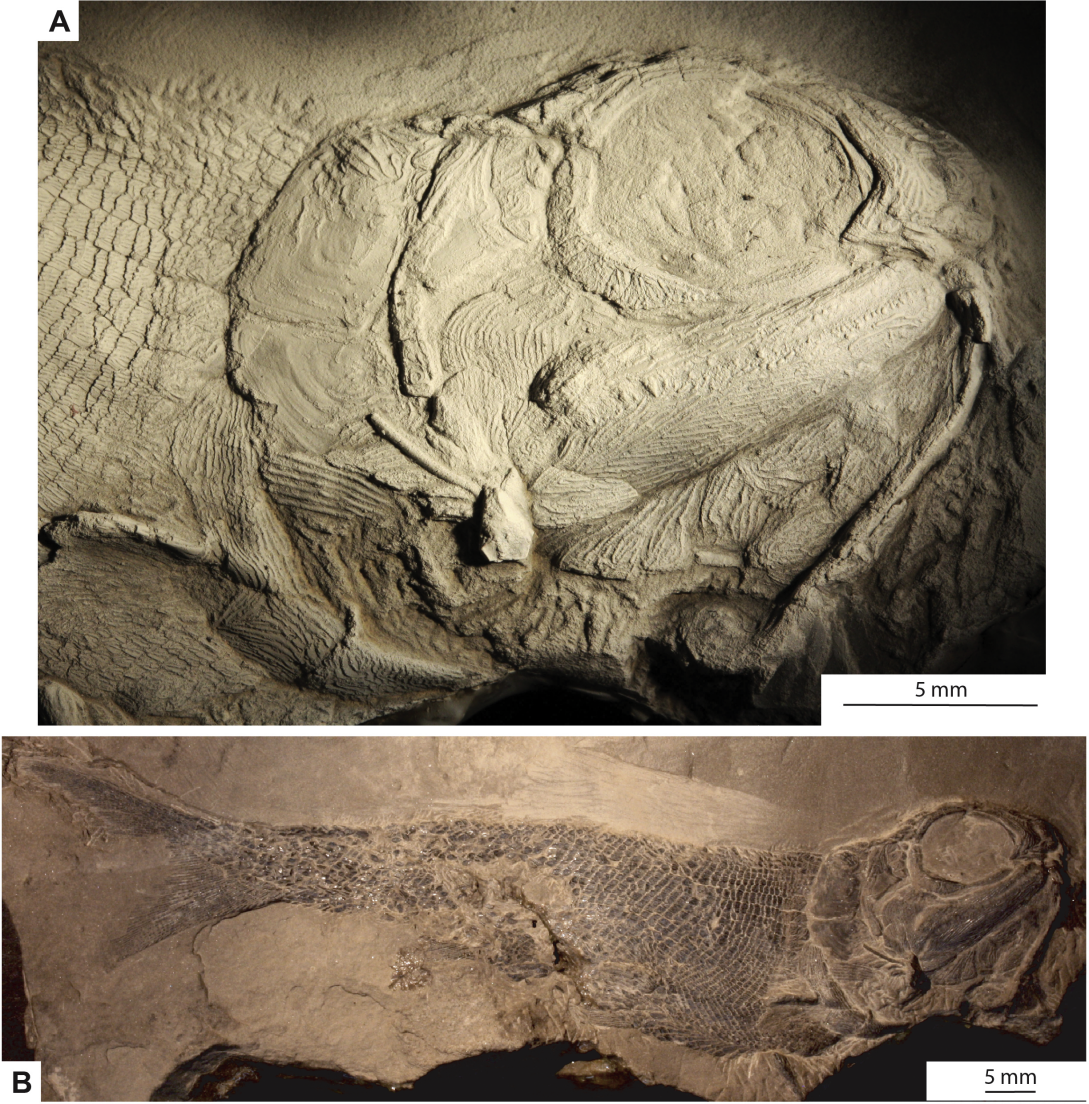
Photographs of the type specimen of* Bluefieldius mercerensis* n. gen. n. sp., KUVP 15584. (A) Lateral view of head. Specimen is shadowed with magnesium oxide. (B) Entire specimen in lateral view. Scale bars equal 5 mm. Photographs were taken by K Mickle.

**Figure 4:**
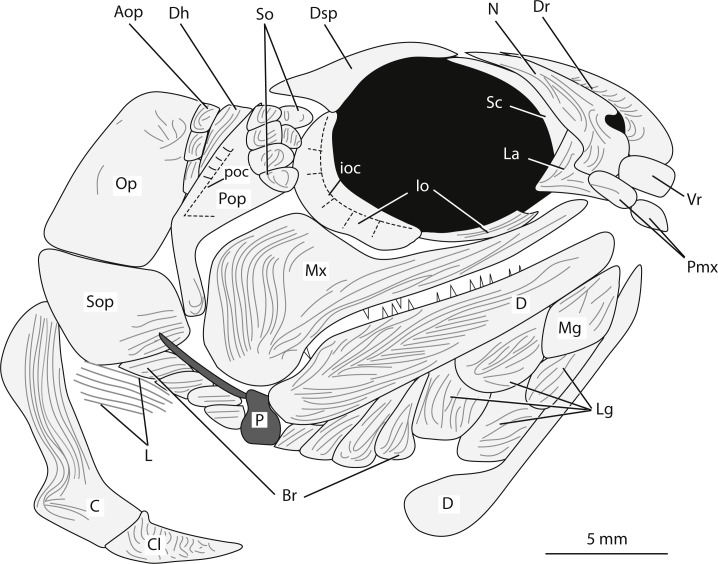
Reconstruction of the head of *Bluefieldius mercerensis* n. gen. n. sp. based on the type specimen KUVP 155843. Illustration details bones and ganoine ornamentation. Abbreviations: Aop, anteopercular bones; Br, branchiostegal rays, C, cleithrum; Cl, clavicle; D, dentary; Dh, dermohyal; Dr, dorsal rostral; Dsp, dermosphenotic; ioc, infraorbital canal; Io, infraorbital; L, lepidotrichia of pectoral fin; La, lacrimal; Lg, lateral gular; Mx, maxilla; Mg, median gular; N, nasal; Op, operculum; P, propterygium; Pmx, premaxilla; Pop, preoperculum; poc, preopercular canal; Sc, sclerotic; So, suborbital; Sop, suboperculum; Vr, ventral rostral. Dashed black lines represent sensory canals, light grey lines represent ganoine ornamentation, and dark grey infilled structures represent endoskeletal elements. Scale bar equals 5 mm.

**Figure 5:**
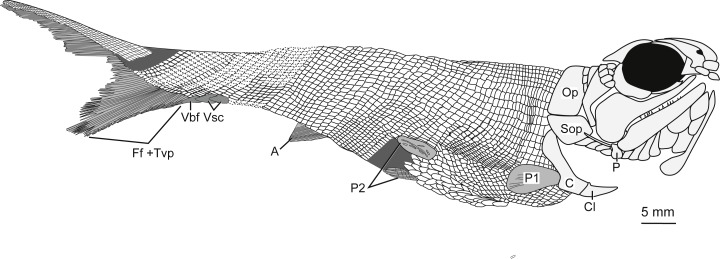
Full body illustration of *Bluefieldius mercerensis* n. gen. n. sp. based on the type specimen KUVP 155843. Dark grey infilling represents areas of disturbance and uncertainty. Abbreviations: A, anal fin; C, cleithrum; Cl, clavicle; Ff +Tvp, fringing fulcra and tips of procurrent rays; Op, operculum; P, propterygium; P1, pectoral fin; P2, pelvic fins; Sop, suboperculum; Vbf, ventral basal fulcrum; Vsc, ventral scutes. Remaining cranial features identified in Fig. 4. Dotted lines are areas scale rows have been reconstructed. Scale bar equals 5 mm.

**Figure 6:**
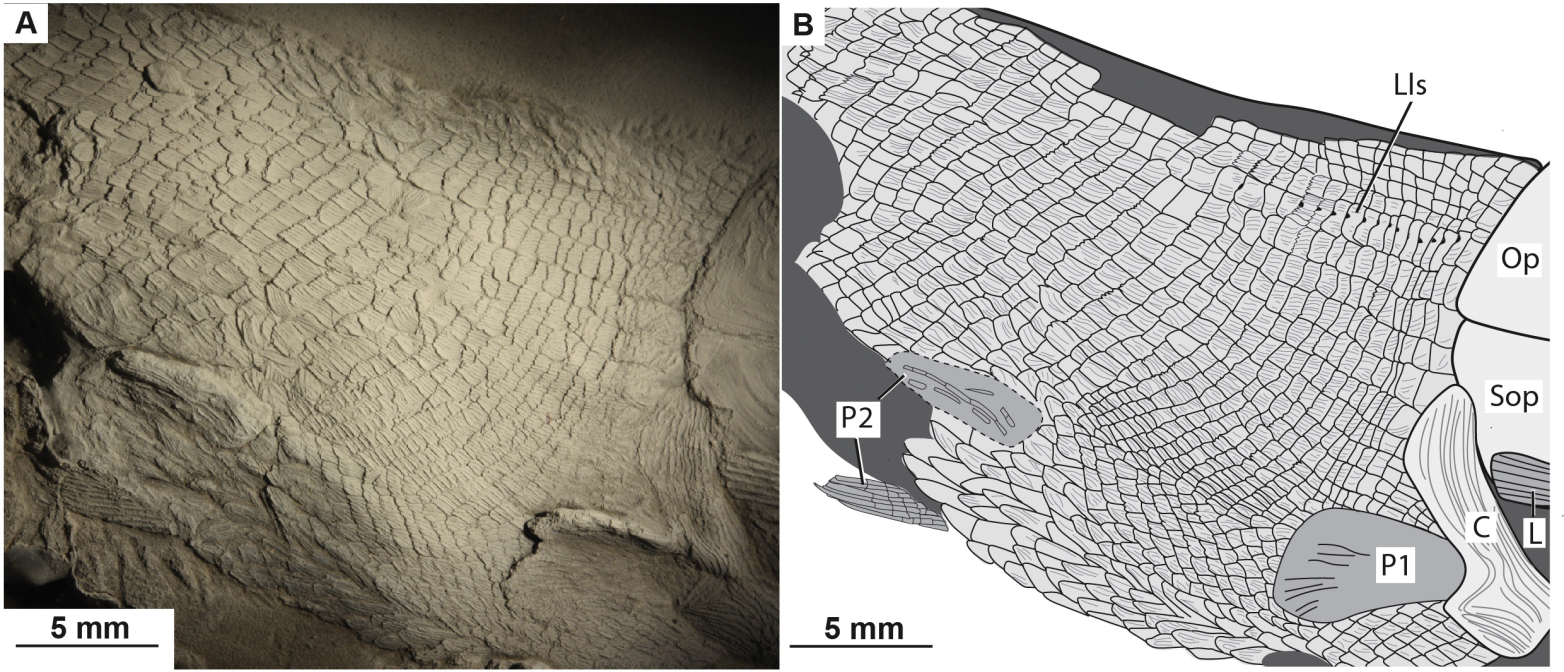
The abdominal region of the type specimen of *Bluefieldius mercerensis* n. gen. n. sp. detailing the pelvic fins and body scales. (A) photograph of KUVP 155843 shaded with magnesium oxide. (B) illustration of KUVP 155843 identifying postcranial features. Abbreviations: C, cleithrum; L, lepidotrichia of pectoral fin; Lls, lateral line scales; Op, operculum; P, propterygium; P1, pectoral fin; P2, pelvic fins; Sop, suboperculum. Dashed line represents margins of pelvic fin, dark grey infilling represents areas of disturbance where scales cannot be reconstructed. Scale bars equal 5 mm. The photograph was taken by K Mickle.

**Figure 7:**
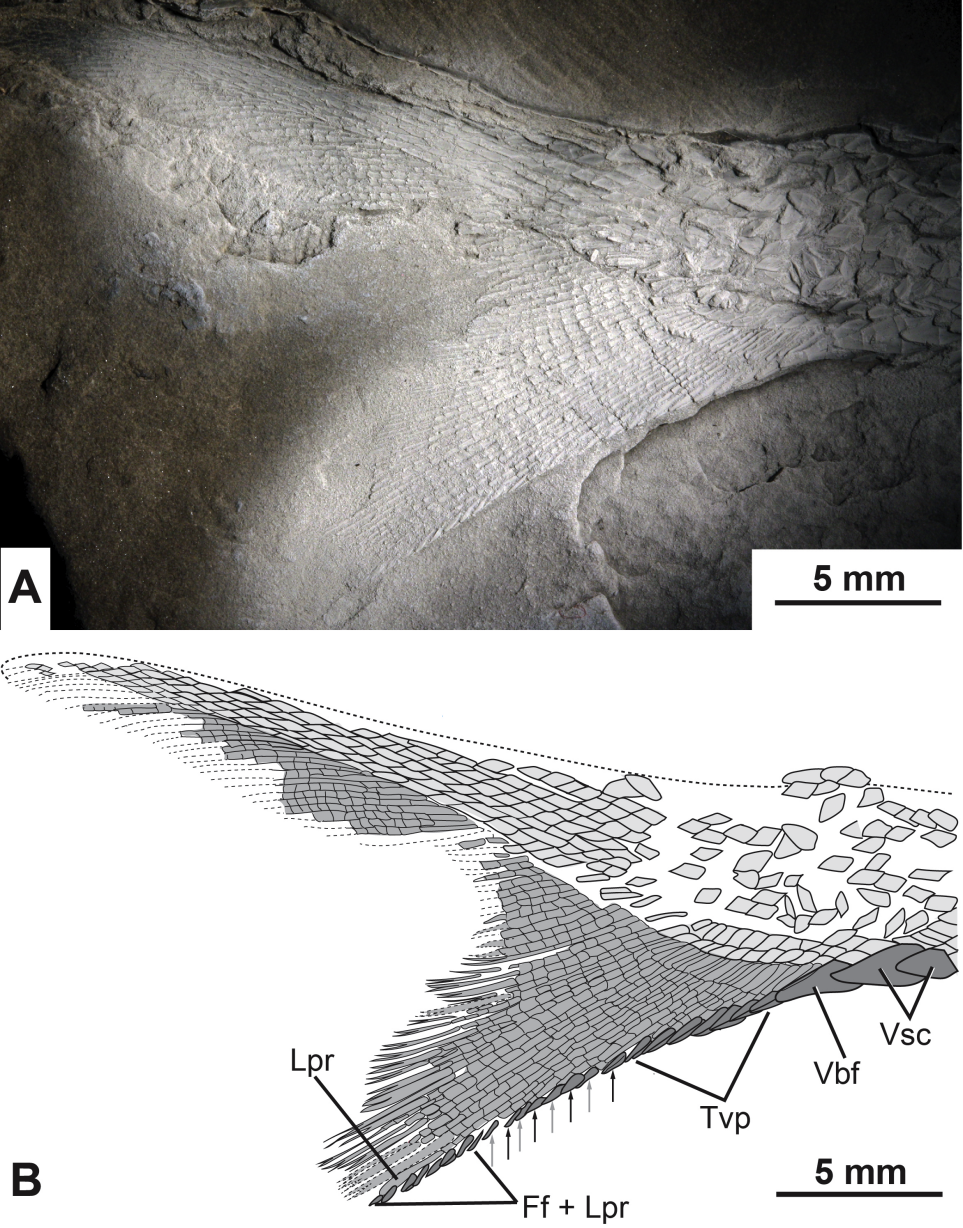
The caudal fin of the type specimen of *Bluefieldius mercerensis* n. gen. n. sp. (A) Photograph of KUVP 155843 shadowed with magnesium oxide. (B) illustration of KUVP 155843 detailing scutes, procurrent fin rays, last principal fin ray, and segmentation and bifurcation of principal fins rays. Abbreviations: Ff + Lpr, fringing fulcra on ventral margin of last principal fin ray; Lpr, last principal fin ray; Tvp, tips of ventral procurrent rays; Vbf, ventral basal fulcrum; Vsc, ventral scutes. Black and grey arrows signify the area where fringing fulcra (black arrows) are inserted between tips of ventral procurrent rays (grey arrows). Dashed lines represent extent of lepidotrichia that are not well enough preserved to detail segmentation and reconstructed dorsal body margin. Scale bars equal 5 mm. The photograph was taken by K Mickle.

*Bluefieldius mercerensis* n. gen. n. sp. (Figs. 3– 7)
urn:lsid:zoobank.org:act:6446F5C9-1A45-424F-9513-0C402FBC06CB

**Etymology.** *mercerensis* in reference to Mercer County, West Virginia where the specimen was recovered.

**Diagnosis.** Based on the unique combination of the following characters: Absence of complex bones in the snout and the presence of distinct and separate lacrimal, premaxillary, and dorsal and ventral rostral bones; narrow rectangular infraorbital ventral to the orbit; large crescent shaped infraorbital posterior and posteroventral to the orbit that contacts a single Y-shaped dermosphenotic dorsally; six small rectangular suborbital bones arranged in two distinct rows; wedge-shaped dermohyal posterior to a hatchet-shaped preoperculum; a row of three rectangular, ganoine-bearing anteopercular bones that extend down half the depth of the operculum; rectangular anteriorly inclined operculum with a diagonal ventral margin, suboperculum that is taller posteriorly than anteriorly and shorter in height but wider in length than the operculum; maxilla with a deep posterior plate, a rounded posteroventral process, and a narrow anterior arm that extends to the anteroventral margin of the orbit; a single median gular, two pairs of lateral gulars, a series of at least eight branchiostegal rays; inequilobate and deeply cleft heterocercal caudal fin with an elongated caudal peduncle; anteriorly placed and mid-body scales with pectinated posterior margins and diagonal ridges of ganoine.

**Holotype and only specimen**. KUVP 155843 (Figs. 3– 7). The holotype and only specimen, KUVP 155843, preserves a nearly complete elongate fusiform fish in lateral view (Figs. 3– 5). The counterpart is missing. The type specimen has a standard length of 8.4 cm and distance from the snout to the fork of the caudal fin of 8.7 cm (FORK of Lund & Poplin, 1997). The caudal peduncle has a depth of 0.7 cm (CPW of Lund & Poplin, 1997) and is elongated. The anal and pelvic fins are nearly complete, remnants of the pectoral fin are present, and the dorsal fin is not preserved (Fig. 3B). Although primarily intact, the skull bones have been disarticulated and separated from the bones of the pectoral girdle (Figs. 3A,  4). The snout is slightly disarticulated, upturned, and preserved with some three-dimensionality. Because of the angle at which the head is upturned, a nearly complete view of the branchiostegal and gular region is preserved but there is no information regarding the bones of the skull roof or the otic region (Figs. 3A,  4).

**Type locality.** Upper Mississippian Bluefield Formation of West Virginia, collected from a road cut off of the north side of highway US 460, in Mercer County, near the border of West Virginia and Virginia, USA. (Fig. 2).

### Anatomical description

**Snout.** The snout region is preserved with some three-dimensionality and is composed of nasal, dorsal rostral, ventral rostral, premaxillary, and lacrimal bones (Figs. 3A,  4). The nasal bone is widest ventrally and tapers to a point dorsally. The nasal bone does not contact the anterior tip of the dermosphenotic; there is a small gap between these two bones that may be an artifact of preservation. Posteroventrally, the nasal bone has a gentle concave margin that contributes to the anterior margin of the lateral/posterior narial opening. Anteroventrally, the nasal is dramatically notched to form the lateral margin of the medial/anterior narial opening. The nasal bone bears vertical ridges of ganoine, except anteroventrally near the narial notches. Here are curved ridges of ganoine that follow the curve of the notch. There is no sign of the supraorbital canal on the nasal bone, but this is likely because of heavy ganoine ornamentation. Posterior to the nasal bone, a sclerotic bone is visible in the anterior portion of the orbit (Figs. 3A,  4). This is the only sclerotic bone preserved.

A single median dorsal rostral bone is present (Figs. 3A,  4). It bears a notch on its posteroventral margin. This notch contributes to the medial margin of the medial/anterior narial opening. The dorsal rostral bone is wider ventrally than dorsally. The majority of the dorsal rostral bone bears horizontal ridges of ganoine, the exception is near the notch that contributes to the narial opening. Here, like the nasal bone, the dorsal rostral bone bears curved ridges of ganoine. Ventral to the dorsal rostral bone is a rectangular ventral rostral bone (Figs. 3A,  4). Though the ethmoid commissure is not seen in this bone, there is a clear border between this bone and the larger dorsal rostral bone, corroborating the identification of this rectangular bone as a ventral rostral. Sensory canal lines are not seen in any of the snout bones, potentially because of the heavy ganoine ornamentation.

Posteroventral to the nasal bone is a separate and distinct lacrimal bone (Figs. 3A,  4). The lacrimal is roughly L shaped with a horizontal arm of bone that extends ventral to the nasal and a vertical portion that is situated posteroventral to the nasal bone (Figs. 3A,  4). The lacrimal is displaced anteriorly so that there is no contact between the lacrimal and the infraorbital bone ventral to the orbit. Because of this disarticulation, the narrow vertical arm of the lacrimal is preserved within the posterior notch in the nasal bone. This is not necessarily the natural contact of the lacrimal and nasal bones. Most likely, the posterior limit of the lacrimal came into contact with anterior margin of the infraorbital ventral to the orbit. The vertical arm of the lacrimal bone may not have been housed within the posterior nasal opening, but in the absence of other non-disturbed specimens, the articulation of the lacrimal bone with the nasal cannot be determined with any confidence. The lacrimal bears vertical ridges of ganoine on the vertical arm of bone and horizontal ridges on the horizontal arm (Figs. 3A,  4).

Anterior to the lacrimal bone and ventral to the nasal and ventral rostral bones lies a small rectangular premaxilla (Figs. 3A,  4). Though teeth are not seen on this bone, this bone is identified as a premaxilla because of its position and relationships to the other bones of the snout. Also visible is the premaxilla from the left side of the fish. The paired premaxillae meet in midline and lie ventral to the nasal and ventral rostral bones.

**Circumorbital series.** Ventral to the orbit, there is a long and narrow infraorbital bone (Figs. 3A,  4). Posteriorly, the narrow infraorbital contacts a large crescent shaped infraorbital (Figs. 3A,  4). The crescent shaped infraorbital extends from the posteroventral corner of the orbit through the entire posterior border of the orbit. The posteroventral margin of this bone has a shallow concavity at about the midpoint of the infraorbital. The posterodorsal corner of the orbit is bound by a Y-shaped dermosphenotic (Figs. 3A,  4). A long narrow arm of the dermosphenotic extends anteriorly along the dorsal margin of the orbit. Posteroventrally, the dermosphenotic has two arms; the posterior-most arm is longer than the anterior-most arm. The anterior-most arm contacts the large crescent shaped infraorbital bone. Between these anterior and posterior-most arms of the dermosphenotic there are two small suborbital bones (Figs. 3A,  4). Traces of the infraorbital canal are preserved on the anterior margin of the large crescent shaped infraorbital. Numerous short canals radiate off of the posterior margin of the infraorbital canal in this bone (Figs. 3A,  4). The narrow infraorbital bone ventral to the orbit bears horizontal ridges of ganoine.

**Skull roof and otic region.** Because of the angle at which the head is upturned, there is no information regarding the bones of the skull roof or otic region.

**Cheek.** The preoperculum is hatchet shaped with a narrow vertical region that extends posterior to the maxilla and an expanded region that lies on the dorsal margin of the maxilla (Figs. 3A,  4). The horizontal pit line is seen in the expanded region. A small section of the preopercular canal is seen in the expanded region, posterodorsal to the horizontal pit line (Figs. 3A,  4). Short canals branch off the posterior margin of the preopercular canal in this region. The preopercular angle is 31°. The majority of the preoperculum is smooth and bears little ganoine sculpturing—there are only a few short ridges of ganoine present on the narrow vertical portion of the preoperculum and the expanded anterodorsal margin near the suborbital bones.

There are six small suborbital bones filling the space between the preoperculum, dermosphenotic, and the crescent shaped infraorbital (Figs. 3A,  4). There is a row of four rectangular suborbital bones that directly contact the preoperculum. Anterior to this row of four bones is an additional row of two suborbital bones that are situated within the curved arms of the dermosphenotic. The suborbital bones that do not contact the preoperculum are smaller than those in contact with the preoperculum. The suborbital bones bear concentric curved ridges of ganoine.

Posterior to the preoperculum is a wedge-shaped dermohyal (Figs. 3A,  4). Dorsally the dermohyal has a rounded margin; ventrally, it narrows to a point. The dermohyal bears ridges of ganoine that run down the anterodorsal length of the bone. Three rectangular anteopercular bones are situated posterior to the dermohyal and anterior to the operculum (Figs. 3A,  4). The anteopercular bones extend past the ventral tip of the dermohyal to about half the height of the operculum. Distinct curved and vertical ganoine ridging is seen on each anteopercular bone.

The maxilla is a large bone with a slender anterior arm that extends to the anteroventral margin of the orbit, a deep and rounded posterior plate, and a rounded posteroventral process (Figs. 3A,  4). The long narrow arm of the maxilla bears close-set horizontal ridges of ganoine whereas the posterior plate bears curved ridges of ganoine. There are large and small conical teeth arranged in two rows on the oral rim of the maxilla. There are teeth along the margin of the posteroventral process. The large conical teeth are vertically oriented on the anterior portion of the maxilla, but on the posterior portion of the maxilla, the large teeth are curved.

**Lower jaw.** The lower jaw is slightly deeper posteriorly and narrows to a rounded point anteriorly (Figs. 3A,  4). The lower jaw bears heavy ganoine ridges in a cantilever pattern of concentric V-shaped ridges posteriorly and horizontal ridges anteriorly. It is not possible to distinguish the individual bones that make up the lower jaw. Vertically oriented conical teeth are present along the oral rim of the lower jaw. Because of the upturned angle of the head, there is evidence of the lower jaw from the left side of the body preserved on this specimen.

**Operculo-gular apparatus.** The operculum is an anteriorly inclined rectangular shaped bone located posterior to the anteopercular bones and the dermohyal (Figs. 3A,  4). It has a width of 4.7 mm and is at an angle of 43°. The ventral margin of the operculum is horizontal and slightly convex. The operculum does not bear ganoine ornamentation aside from a few diagonal ridges on the anterodorsal corner of the bone. Smooth, concentric blocks in the shape of the operculum are seen increasing in size and may represent growth rings. The same growth rings are seen on the suboperculum. The suboperculum has a diagonal dorsal margin and is taller posteriorly than anteriorly (Figs. 3A,  4). The suboperculum is less than half the opercular depth but it is wider than the operculum. The dorsal margin is slightly concave and the convex ventral margin of the operculum is situated in this concavity.

At least eight branchiostegal rays are present (Figs. 3A,  4). Each branchiostegal ray bears ridges of ganoine. A single median gular is present as a large, ovoid bone that tapers to rounded points anteriorly and posteriorly. There are two pairs of ovoid lateral gulars posterior to the median gular that are of similar size and shape to the median gular (Figs. 3A,  4). The second set of lateral gulars are identified as gulars rather than branchiostegal rays because they are more similar in size and shape to the anteriorly placed set of lateral gulars than branchiostegal rays. The first pair of branchiostegal rays posterior to the lateral gulars, and the subsequent branchiostegal rays, are narrower and more rectangular in shape than the lateral gulars (Figs. 3A,  4).

**Pectoral girdle.** Elements of the pectoral girdle have been separated from the anteriorly placed bones of the head creating an area of rocky matrix between the pectoral girdle bones and the cranial bones (Figs. 3,  4). The posterior margin of the cleithrum is deeply notched midway down the bone (Figs. 3A,  4). Ventral to this notch, the cleithrum abruptly increases in width. Dorsal to the notch, the width of the cleithrum gradually increases. The dorsal most margin of the cleithrum is obscured at around the midpoint of the suboperculum. The majority of the cleithrum bears long vertical ridges, but a few curved ridges are seen at the level of the notch. A triangular clavicle is present (Figs. 3A,  4). When viewed laterally, this bone is elongated and drawn out anteriorly into a pointed tip. There is no information regarding a posttemporal or supracleithrum preserved in KUVP 155843.

**Squamation.** There are robust large rectangular post-cleithral scales. These scales are found in the first row posterior to the pectoral girdle and the suboperculum (Figs. 3,  5–6). The anteriorly placed flank scales are rectangular and have pectinated posterior edges, with about six to seven pectinations per scale (Fig. 6). The scales bear horizontal ridges of ganoine, and these ridges end in pectinations. The anterodorsally placed scales are smaller than the anteriorly placed flank scales, but they are also pectinated and bear horizontal ridges of ganoine. The anteroventrally placed scales are shorter and more rectangular in shape than the flank scales. Because of how the fish is preserved, the scales on the ventral side of the fish are visible—these scales, especially between the pelvic fins, are long and diamond shaped (Fig. 6).

The scales carrying the lateral line canal are identifiable by the notch in the middle of their posterior margins (Fig. 6). There is no ganoine ornamentation at the level of these notches, but there are horizontal ridges of ganoine dorsal and ventral to these notches (Fig. 6).

Dorsally placed mid body scales are diamond shaped, but have rounded convex ventral margins. These scales bear about three to four serrations per scale (Fig. 6). A slight anterodorsal expansion is seen on some of these scales. Mid body flank scales are shorter than the anteriorly placed flank scales. These flank scales are also serrated, but with five serrations per scale, there are fewer serrations than on the anteriorly placed flank scales. Ventral mid body scales are short and bear about three serrations.

Scales placed along the caudal peduncle do not show the same degree of ganoine sculpturing as the more anteriorly placed scales. Posteriorly placed scales are diamond shaped and smooth with no ganoine ornamentation. The scales along the caudal peduncle are much shorter than all of the other scales seen on the body.

**Fins.** The right pectoral fin is *in situ* and visible posterior to the notch in the cleithrum (Figs. 3,  5). It is difficult to count the individual lepidotrichia of the pectoral fin because of its positioning above scales and preservation. Endoskeletal elements of the pectoral girdle are preserved near the area of dislocation of the skull from the pectoral girdle bones (Figs. 3A,  4). There is a three dimensional bulge with a long tubular extension off of its posterodorsal margin. This element (Figs. 3A,  4) resembles the propterygium and leading fin ray described by Gardiner (1984) in *Moythomasia durgaringa.* There is no evidence of a canal on the propterygium in this specimen. Close to the propterygium, ventral to the suboperculum, posteroventral to the first few branchiostegal rays, and dorsal to cleithrum is a series of horizontal elements that are identified here as lepidotrichia (Figs. 3A,  4, 7). These lepidotrichia are not segmented, suggesting they are anterior lepidotrichia of the pectoral fin, similar to the non-segmented sections of the pectoral fin lepidotrichia of *Gogosardinia coatesi* (Choo, Long & Trinajstic, 2009), *Mimipicis* (Gardiner, 1984; Choo, 2011), *Howqualepis rostridens* (Long, 1988, figs. 27–28, Choo, 2011), and *Melanecta anneae* (Coates, 1988).

The dorsal fin is not preserved. There are remnants of an anal fin with fringing fulcra on its anterior border (Figs. 3B, 5). The number of fin rays in the anal fin is 12 and the snout to anal fin origin measurement (SAO of Lund & Poplin, 1997) is 5.6 cm. Because of the positioning of the body, both pelvic fins are visible (Figs. 3B,  5–6). The most complete and visible pelvic fin has 14 fin rays.

A relatively complete heterocercal caudal fin is preserved with a base length of 2.8 cm (CBAS of Lund & Poplin, 1997). The lepidotrichia are tightly packed, highly segmented, and the majority of the principal fin rays are distally bifurcated (Fig. 7). Scutes and fulcra associated with the hypaxial lobe of the caudal fin are well preserved. There are three unpaired elements anterior to the ventral lobe of the caudal fin that overlap each other so that the anterior margins of the second and third elements are covered by the posterior portions of the first and second elements, respectively (Fig. 7). The first two unpaired elements are identified as ventral scutes because of their horizontal orientation to the main body axis and their placement anterior to the fin rays (Fig. 7). The third element is identified as a ventral basal fulcrum because of its more oblique orientation with respect to the main body axis and its closer association to the fin rays (Fig. 7).

Following the single ventral basal fulcrum is a series of 11 segmented, yet unbranched procurrent fin rays. Each of the procurrent fin rays ends distally with a short pointed fulcrum-like tip (Fig. 7).

Posterior to the series of procurrent rays are principal rays. The first fin ray directly following the hypaxial procurrent fin rays is segmented but not branched distally. This element is identified as the last distal principal ray of the caudal fin. Principal fin rays above this unbranched last ventral principal ray are bifurcated distally. The first segmented but unbranched principal ray associated with the epaxial lobe of the caudal fin is not visible.

Fringing fulcra are present along the hypaxial lobe of the caudal fin on the ventral margin of the unbranched last principal fin ray, and in some areas, inserted between the fulcra-like tips of the procurrent rays. The last eight structures along the ventral margin of the caudal fin are fringing fulcra (Fig. 7). These eight elements are found on the ventral margin of the last unbranched principal ray (Fig. 7). Anterior to the fringing fulcra along the last principal ray is a series of fringing fulcra inserted between the tips of sequential procurrent rays (Fig. 7). In this alternating series, there is one fringing fulcrum (Fig. 7, black arrows) inserted between the tips of two sequential fin rays (Fig. 7, grey arrows). There are a total of four inserted fringing fulcra (Fig. 7). Anterior to the series of inserted fringing fulcra is the series composed of the fulcra-like tips of procurrent rays (Fig. 7). Collectively, the fringing fulcra and procurrent fin ray tips line the ventral margin of the caudal fin (Fig. 5, Fig. 7). Epaxial scutes and fulcra of the caudal fin are either not preserved or so fine that details cannot be discerned.

## Discussion

### Osteological features of note and comparisons to previously described fishes

*B. mercerensis* n. gen. n. sp. differs from other Carboniferous fishes in specific cranial characteristics. The combination of the presence of lacrimal and premaxillae that do not contribute to the formation of complex bones, a large crescent shaped infraorbital in the posteroventral corner of the orbit which contacts the dermosphenotic, and multiple suborbital and anteopercular bones are some of the features that make *B. mercerensis* n. gen. n. sp. unique and distinct from other Carboniferous actinopterygians and support the description of a new genus. The unique characteristics of *B. mercerensis* n. gen. n. sp. are especially apparent when this new taxon is compared to other Late Carboniferous North American fishes such as the well-preserved Serpukhovian actinopterygians from the Bear Gulch Limestone of Montana, USA. The Bear Gulch Limestone actinopterygians are Late Mississippian (Serpukhovian, Namurian E2b) in age and are good comparisons for this newly described fish. The condition of the bones of the snout, infraorbitals series, suborbitals, and anteopercular bones in *B. mercerensis* n. gen. n. sp. is compared to that of Bear Gulch actinopterygians and other Carboniferous fishes in the sections below to highlight how this new taxon is distinct from previously described actinopterygians.

**Figure 8:**
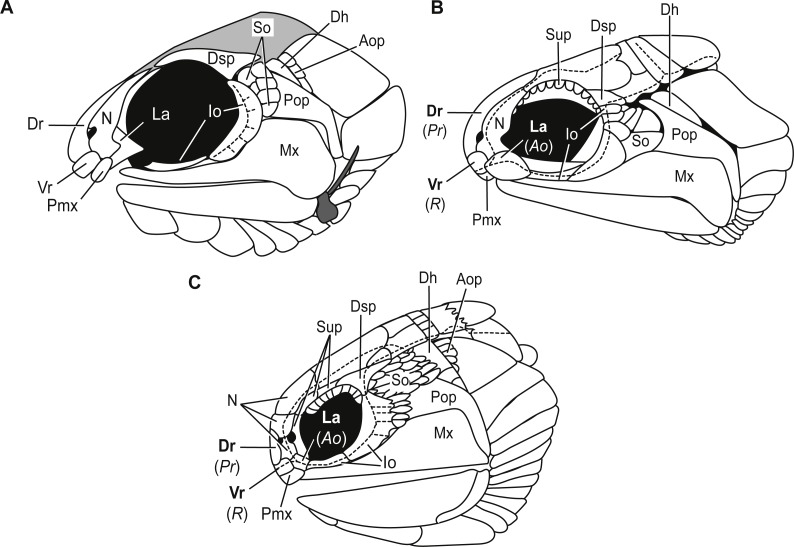
Comparative illustrations of fishes with dorsal rostral, ventral rostral, premaxillary, and lacrimal bones highlighting similarities and differences of skull bones between these taxa. (A) Reconstruction of *B. mercerensis* n. gen. n. sp. based on KUVP 155843. Illustration reflected, premaxilla from opposite side removed for simplicity. This reconstruction rearticulates bones posterior to the snout region but the lacrimal is left disarticulated because of uncertainty of the in situ condition of this bone. The grey infilled area is a reconstruction of the extent of the skull roof. (B) *Kalops monophrys* modified from (2002, fig. 2). (C) *Paratarrasius hibbardi* modified from Lund & Melton (1982, fig. 1A) and Lund & Poplin (2002, fig. 1D). Identifications of the bones of the snout have been updated from the original descriptions of *Kalops* and *Paratarrasius* to use the terminology of Mickle (2012) and Mickle (2015). Bone names that have changed are signified in bold typeset. The original identifications are given below the updated names in parentheses and italics. Abbreviations: Ao, antorbital; Aop, anteopercular bones; Dh, dermohyal; Dr, dorsal rostral; Dsp, dermosphenotic; Io, infraorbitals; La, lacrimal; Mx, maxilla; N, nasal; Pmx, premaxilla; Pop, preoperculum; Pr, postrostral; R, rostral; So, suborbitals; Sup, supraorbitals; Vr, ventral rostral. Dashed black lines represent sensory canals.

**Bones of the snout.** The snout of Carboniferous lower actinopterygians is characterized by a great deal of anatomic diversity in terms of the numbers and identities of its bones of this region. Carboniferous actinopterygians differ from Devonian fishes in that there are numerous Carboniferous species with separate and distinct lacrimal and premaxillary bones, a condition not seen in Devonian fishes (Mickle, 2015; Mickle, 2017). Lower actinopterygians with separate and distinct lacrimal and premaxillary bones include *Canobius ramsayi* (Moy-Thomas & Bradley Dyne, 1938) and Bear Gulch Limestone taxa within the genera *Kalops* (Poplin & Lund, 2002), *Lineagruan* (Mickle, Lund & Grogan, 2009), and *Beagiscus* (Mickle, Lund & Grogan, 2009). *Bluefieldius mercerensis* n. gen. n. sp. is an example of another Carboniferous actinopterygian with separate lacrimal and premaxillary bones (Figs. 3A,  4). The presence of these bones in combination with ventral rostral and dorsal rostral bones is less commonly described but it is a characteristic shared between *Bluefieldius* n. gen. and the genera *Kalops* and *Paratarrasius* from the Bear Gulch Limestone (Lund & Melton, 1982; Lund & Poplin, 2002; Poplin & Lund, 2002; Mickle, 2015; Fig. 8). Though these genera are defined by the presence of ventral rostral, dorsal rostral, lacrimal, and premaxillary bones, they are distinct and different from each other (Fig. 8). The elongated tarrasiid has many anatomical characteristics of the skull (numerous nasal bones (Fig. 8), presence of supraorbital bones, and shape of maxilla), body shape (elongate), and fins (continuous unpaired fins, lack of pelvic fins) that are not seen in *Bluefieldius* n. gen. (Lund & Melton, 1982; Lund & Poplin, 2002). *Kalops* and *Bluefieldius mercerensis* n. gen. n. sp. differ in the shape of the lacrimal bone (Fig. 8). The lacrimal bone in *Kalops* (Fig. 8) is ovoid and is positioned ventral to the nasal with no vertical extension located posterior to the nasal bone as in *B. mercerensis* n. gen. n. sp. (Poplin & Lund, 2002). In addition *Bluefieldius* n. gen. n. differs from *Kalops* in regard to the absence of supraorbital bones (Fig. 8) and a scaled lobe to the pectoral fin and features of the infraorbital, suborbital, and opercular bones that are discussed below (Poplin & Lund, 2002).

**Infraorbital bones.** *B. mercerensis* n. gen. n. sp. has one large crescent shaped infraorbital bone in the posteroventral corner of the orbit (Figs. 3A,  4, 8). This large bone comes into contact with the dermosphenotic. The presence of a large crescent shaped infraorbital that forms the majority of the posterior border of the orbit is a characteristic *B.* n. gen. shares with many Devonian and Carboniferous genera such as *Mimipiscis* (Gardiner, 1984)*, Moythomasia* (Gardiner, 1984)*, Howqualepis* (Long, 1988), *Mansfieldiscus* (Long, 1988), *Gonatodus* (Gardiner, 1967), *Woodichthys* (Coates, 1988), and the Bear Gulch genera *Wendyichthys* (Lund & Poplin, 1997)*, Cyranorhis* (Lund & Poplin, 1997)*, Lineagruan,* and *Beagiascus* (Mickle, Lund & Grogan, 2009). *Bluefieldius mercerensis* n. gen. n. sp. differs from these Devonian and Carboniferous fishes in regards to the composition of the bones of the snout and other features of the cranial bones. It is important to note that the contact between the large crescent shaped infraorbital and the dermosphenotic is another feature that sets *Bluefieldius* n. gen. apart from the genus *Kalops* (Fig. 8)*. Kalops* has a series of small infraorbital bones separating the crescent shaped infraorbital in the posteroventral corner of the orbit from the dermosphenotic (Poplin & Lund, 2002).

**Suborbital bones.** Suborbitals are anamestic bones that are found posterior to the circumorbital bones, separating these bones from the preoperculum. Suborbital bones are uncommon in Devonian fishes; they are absent in, among others, *Cheirolepis* (Pearson & Westoll, 1979; Arratia & Cloutier, 1996), *Moythomasia durgaringa* (Gardiner, 1984), *Mimipiscis* (Gardiner, 1984), and *Gogosardinia* (Choo, Long & Trinajstic, 2009). The only Devonian fishes known with suborbital bones are *Moythomasia nitida* (Gross, 1953; Jessen, 1968) with one or two suborbital bones and *Osoriochthys marginis* (Taverne, 1997) which has one large suborbital bone (Mickle, 2017). Suborbital bones are seen commonly in Carboniferous actinopterygians in two general patterns. Some Carboniferous fishes such as the Scottish Namurian *Mesopoma carricki* (Coates, 1993) and the Serpukhovian *Wendyichthys* and *Cyranorhis* (Lund & Poplin, 1997), are characterized by the presence of one or two large suborbital bones whereas other Carboniferous fishes are characterized by more than two suborbitals filling the space between the circumorbital bones and the preoperculum. For those with more than two suborbital bones, fishes can be characterized by having three or more medium sized bones, such as the Bear Gulch *Lineagruan* (Mickle, Lund & Grogan, 2009), or a mosaic of smaller bones that may vary in number from individual to individual such as the condition in *Paratarrasius* (Lund & Melton, 1982), *Kalops* (Poplin & Lund, 2002), and *Beagiascus* (Mickle, Lund & Grogan, 2009). *Bluefieldius mercerensis* n. gen. n. sp., with six small suborbital bones (Figs. 3A,  4), is best characterized as having a mosaic of small suborbital bones, though the suborbitals are neatly arranged in two distinct rows in this new taxon.

**Anteoperular bones.** Kazantseva-Selezneva (1982) described three potential conditions for the zone between the opercular apparatus and the cheek in lower actinopterygians. There could be no intervening bones between the cheek region and the opercular apparatus, a single or serial dermohyal, or a dermohyal plus several anteopercular (accessory opercular) bones (Kazantseva-Selezneva, 1982; Mickle, Lund & Grogan, 2009). *Bluefieldius mercerensis* n. gen. n. sp. is characterized by the third condition—there is a series of anteopercular bones posterior to a wedge-shaped dermohyal and anterior to the operculum (Figs. 3A,  4). This condition is seen in Carboniferous fishes including *Lambeia pectinatus* from the Albert Shale Formation (Mickle, 2017) and the Bear Gulch *Paratarrasius* (Lund & Melton, 1982; Lund & Poplin, 2002) and *Lineagruan* and *Beagiascus* (Mickle, Lund & Grogan, 2009). *Bluefieldius* n. gen. differs from *Lineagruan* and *Beagiascus* in the extent of the anteopercular bones. In *B. mercerensis* n. gen. n. sp., the anteopercular bones extend down to about half the height of the operculum; in *Lineagruan, Beagiascus,* and *Lambeia* the anteopercular bones extend down the entire height of the operculum, or even to the ventral margin of the suboperculum (Mickle, Lund & Grogan, 2009; Mickle, 2017). The thickness of these bones vary between *Bluefieldius* n. gen. and *Lineagruan.* The anteopercular bones, though ornamented with ganoine, are thin and delicate in *Lineagruan judithi* whereas the anteopercular bones in *Bluefieldius mercerensis* n. gen. n. sp. are of the same thickness as the surrounding bones, a condition similar to that seen in *Lambeia* (Mickle, 2017). *Paratarrasius* has anteopercular bones that do not extend down to the ventral margin of the operculum (Fig. 8), but these bones are different from those in *Bluefieldius* n. gen. in terms of size, shape, and number (Lund & Melton, 1982; Lund & Poplin, 2002).

## Conclusions

A new lower actinopterygian genus and species is described from the Upper Carboniferous Bluefield Formation of West Virginia. This new genus is represented by a well preserved articulated specimen which represents the first vertebrate body fossil from the Bluefield Formation and the first described actinopterygian. *B. mercerensis* n. gen. n. sp. is defined by a unique set of cranial characteristics including morphological characters of the snout, the circumorbital series, suborbital bones, and anteopercular bones. The combination of these specific characteristics separate *B. mercerensis* n. gen. n. sp. from previously described lower actinopterygian fishes and warrant the description of a new taxon.

Lower actinopterygian fishes are characterized by a great deal of anatomic and taxonomic diversity that is not well understood. We have neither a stable classification scheme nor strongly supported hypotheses of relationships for lower actinopterygian fishes. This lack of understanding has been attributed to the need for more well-preserved fishes to be described or redescribed and a better understanding of morphological characters among lower actinopterygians (Cloutier & Arratia, 2004; Mickle, 2015; Mickle, 2017). Taxonomic work provides opportunities to uncover new morphological characters or to see characters in different light and can lead to reassessments of morphological characters. These taxa and characters are the raw material for phylogenetic analyses and more well described taxa and characters will bring stronger hypotheses of relationships for these important fishes.

## Anatomical Abbreviations

A: anal fin
Ao: antorbital
Aop: anteopercular bones
Br: branchiostegal rays
C: cleithrum
Cl: clavicle
D: dentary
Dh: dermohyal
Dr: dorsal rostral
Dsp: dermosphenotic
Ff + Lpr: fringing fulcra on ventral margin of last principal ray
Ff +Tvp: fringing fulcra and tips of ventral procurrent rays
Io: infraorbital
ioc: infraorbital canal
L: lepidotrichia of pectoral fin
La: lacrimal
Lg: lateral gular
Lls: lateral line scales
Lpr: last principal ray
M: Meramecian
Mx: maxilla
Mg: median gular
N: nasal
Op: operculum
P: propterygium
P1: pectoral fin
P2: pelvic fins
Pmx: premaxilla
Pop: preoperculum
poc: preopercular canal
Pr: postrostral
R: rostral
Sc: sclerotic
So: suborbital
Sop: suboperculum
Sup: supraorbital
Tvp: tips of ventral procurrent rays
Vbf: ventral basal fulcrum
Vr: ventral rostral
Vsc: ventral scutes

## Institutional Abbreviations

KUVP: Division of Vertebrate Paleontology, Natural History Museum and Biodiversity Research Institute, University of Kansas, Lawrence, Kansas, USA.

## Additional Information and Declarations

### Data Availability

The following information was supplied regarding data availability: The type specimen of the newly described taxon is housed within the University of Kansas Natural History Museum (Lawrence, Kansas, USA) in the Vertebrate Paleontology collection with the specimen number KUVP 155843.

### New Species Registration

The following information was supplied regarding the registration of a newly described species: Publication LSID: urn:lsid:zoobank.org:pub:0F2C9D33-9584-4739-B914-5C4C243E0B1F New Genus: Bluefieldius Bluefieldius: urn:lsid:zoobank.org:act:01FDF534-6985-4E62-9CAF-EAE6FB4795B1 New species: *Bluefieldius mercerensis* *Bluefieldius mercerensis*: urn:lsid:zoobank.org:act:6446F5C9-1A45-424F-9513-0C402FBC06CB.
